# Effect of Anchorage Modifications on the Efficacy of Miniscrew-Assisted Rapid Palatal Expansion: A Systematic Review and Meta-Analysis

**DOI:** 10.7759/cureus.72008

**Published:** 2024-10-21

**Authors:** Yury A Villa-Obando, Sandra M Correa-Osorio, Robinson A Castrillon-Marin, Anny M Vivares-Builes, Carlos M Ardila

**Affiliations:** 1 Orthodontics, School of Dentistry, Institución Universitaria Visión de las Américas, Medellín, COL; 2 Orthodontics, School of Dentistry, Institución Universitaria Visión de las Américas, Medellin, COL; 3 Basic Sciences, Faculty of Dentistry, University of Antioquia, Medellin, COL

**Keywords:** maxillary expansion, miniscrews, orthodontic anchorage procedures, palatal expansion technique, randomized controlled trials

## Abstract

Mini-implant-assisted rapid palatal expansion (MARPE) offers a non-surgical alternative for expanding the basal bone, increasing skeletal effects while minimizing undesirable dental side effects. This systematic review and meta-analysis evaluate the effectiveness of MARPE in terms of transverse skeletal development, dentoalveolar changes, and periodontal effects, with consideration of appliance design.

A review was conducted across multiple databases, including PubMed, MEDLINE, Embase, Scopus, and Springer, covering studies published between 2005 and 2024. Randomized controlled trials involving MARPE, with specific attention to design variations (e.g., miniscrew type, number, diameter, length, position, and activation protocol), were selected.

Transverse skeletal expansion, assessed via changes in the nasal width, was reported in six of seven studies, with an average increase ranging from 0.31 mm to 2.90 mm for MARPE, compared to 0.11 mm to 2.46 mm for conventional Hyrax expanders. Maxillary width expansion ranged from 2.89 mm to 9.08 mm with MARPE and from 2.59 mm to 8.51 mm with Hyrax. Periodontal changes were reported in six studies, although details were generally unclear. The use of four self-drilling miniscrews with once-daily activation over an extended period produced greater nasal width expansion, greater average skeletal expansion of the maxilla, and less dental inclination and dentoalveolar expansion compared to Hyrax. No significant differences were observed between short and long miniscrews, although wider miniscrews resulted in improved outcomes.

The MARPE technique, especially when using a design with four self-drilling miniscrews and daily activation until the targeted expansion is reached, offers notable benefits compared to the conventional Hyrax appliance. This method improves both nasal and maxillary skeletal expansion, lessens dental inclination, and reduces negative effects relative to traditional approaches.

## Introduction and background

Transverse deficiencies or alterations are conditions of multifactorial etiology that often manifest in early childhood and can present in various forms. Several orthopedic and surgical techniques have been developed to address transverse discrepancies, with maxillary expansion being one of the most widely used since it was first described by Emerson C. Angell [1]. This technique has been employed in patients with maxillary growth deficits, crossbite, cleft lip and palate, and as part of preoperative procedures for orthognathic surgery or functional jaw orthopedics [2].

Maxillary expansion appliances can be either fixed or removable, and various anchorage approaches have been utilized, including tooth-borne, tooth-tissue-borne, and now exclusively bone-borne anchorage. Among the appliances commonly used in maxillary orthopedics and orthodontics are the Hawley plate with expansion screws, the quadhelix fixed appliance, and for rapid maxillary expansion (RME), devices like the Hyrax and Hass-type expanders. These appliances work by separating the midpalatal suture and activating the maxillary sutural system. Although these devices fell out of favor for some time, likely due to a lack of robust evidence supporting their benefits [3], advances in bone-anchored techniques have led to their resurgence, particularly with the use of titanium implants, which provide absolute anchorage without requiring patient cooperation.

Today, these appliances represent the most reliable means of correcting transverse maxillary deficiencies by applying lateral force through the expander to separate the two halves of the palatal bone. This separation typically follows a triangular pattern, with the apex towards the nasal cavity and the base at the level of the palatine process, resulting in greater anterior than posterior expansion of the midpalatal suture [4].

**Figure 1:**
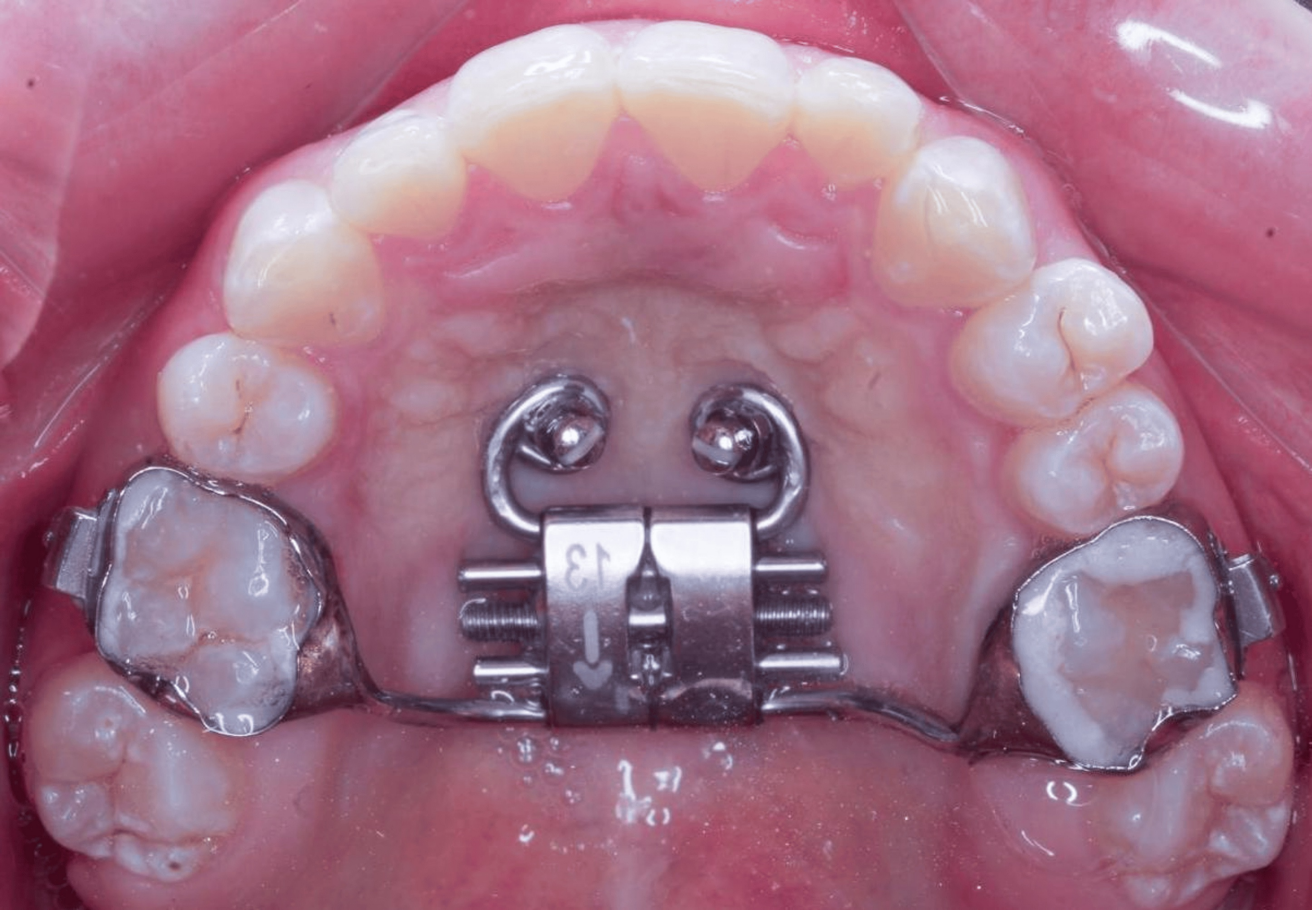
MARPE device in position MARPE: Mini-implant-assisted rapid palatal expansion Image credit: Robinson A. Castrillon-Marin

Mini-implant-assisted rapid palatal expansion (MARPE) offers a non-surgical alternative for expanding the basal bone, increasing skeletal effects while minimizing undesirable dental side effects. By using mini-implants, miniscrews, or temporary anchorage devices (TADs), MARPE achieves absolute anchorage and more controlled expansion [5]. Figure 1 shows a MARPE device in position.

To date, several systematic reviews have evaluated MARPE, focusing on its efficacy, comparing tooth-borne versus bone-borne RME, or examining its use in adult patients approaching sutural fusion. However, none have specifically evaluated its effectiveness in terms of transverse skeletal development or its dentoalveolar and periodontal effects, considering appliance design variables such as the number, diameter, and length of screws, the placement of mini-implants, or modifications to the expander and activation protocols.

This gap in the literature highlights the need for more evidence-based approaches to MARPE design and use. It is essential for orthodontists to have access to scientifically validated information that can guide clinical decision-making regarding appliance design, placement, and activation to optimize patient outcomes. Thus, the objective of this systematic review and meta-analysis is to evaluate the effectiveness of MARPE in terms of transverse skeletal development, dentoalveolar changes, and periodontal effects, with a particular focus on appliance design.

## Review

Materials and methods

This systematic review was conducted following the Preferred Reporting Items for Systematic Reviews and Meta-Analyses (PRISMA) guidelines [6]. The protocol was registered in the International Prospective Register of Systematic Reviews (PROSPERO) record CRD42021256989.

Search Strategy and Selection Criteria

Two authors independently conducted the selection of studies published between January 2005 (the start date of the MARPE technique) [3-5] and May 2024, reporting on the efficacy of MARPE treatment. A systematic search was performed using electronic databases (PubMed, MEDLINE, Embase, Scopus, Springer), as well as a manual search of gray literature through Google Scholar, the CAPES database (Brazil), and the System for Information on Grey Literature in Europe (SIGLE). No language restrictions were applied.

To retrieve eligible studies, a search strategy was initially developed for PubMed and then adapted for the other databases. Both DeCS and MeSH terms were used where possible, along with free-text descriptors, including: "Rapid palatal expansion" OR "rapid maxillary expansion" OR "transpalatal expansion" OR "MARPE" OR "miniscrew-assisted rapid maxillary expansion" OR "microscrew-assisted rapid maxillary expansion" OR "miniimplant-assisted rapid maxillary expansion" OR "TAD-assisted rapid maxillary expansion" OR "transverse maxillary expansion" (free-text descriptors); "exp orthodontics, corrective/" OR "exp orthodontics, interceptive/" OR "(expand and (appliance OR device))" OR "(transpalatal OR trans-palatal) and palatal expansion technique" (MeSH); "bone-borne" OR "bone-anchored" OR "transpalatal expander" OR "palatal expander" OR "hybrid hyrax" OR "skeletally-anchored" (free-text descriptors).

The exclusion criteria included studies that involved expansion combined with other treatments, surgical procedures, or patients with cleft lip/palate, craniofacial syndromes, or systemic conditions. Animal studies, in vitro studies, review articles, case studies, case series, editorials, and expert opinions were also excluded.

Study Screening and Data Extraction

Studies were screened sequentially by title, abstract, and full text. Reference lists of the selected studies were reviewed to identify additional relevant articles.

The following data were extracted for study characterization: author, year of publication, study design, objective, country, sample size, methodology, average age, type of transverse maxillary deficiency, observed measurements (skeletal, dentoalveolar, and/or periodontal), follow-up duration, measurement methods, and main outcomes.

For articles related to MARPE design, the extracted data included: author, year of publication, miniscrew type, length (mm), diameter (mm), position in the arch, activation protocol, and treatment duration (in weeks).

Cohen's kappa coefficient was used to assess inter-reviewer agreement, with near-perfect agreement achieved (kappa value of 0.89) [7].

*Quality and Risk of Bias Assessmen*t

Quality was assessed using the Downs and Black scale, which includes 28 points distributed across 27 questions and five subscales (reporting, external validity, internal validity, confounding, and power) [8]. For the risk of bias, the Cochrane Collaboration's tool was used [9]. Graphics were generated using the robvis tool [10].

A study was classified as having an overall low risk of bias if only one criterion was evaluated as unclear. If two criteria were evaluated as unclear, the study was classified as having an unclear risk of bias. If more than two criteria were evaluated as high risk, the study was classified as having an overall high risk of bias.

Data Synthesis and Analysis

Data were synthesized qualitatively by summarizing findings from studies classified as having an overall low risk of bias and providing high-quality or excellent evidence.

A qualitative synthesis was also conducted to identify modifications in MARPE appliance design. Meta-analyses were performed to identify key design characteristics that optimize MARPE effectiveness, with a focus on maximizing benefits and minimizing undesirable effects. Statistical analyses were performed using R 4.2.1 (R statistical software; R Foundation for Statistical Computing, Vienna, Austria), a free software environment for statistical computing.

Results

**Figure 2:**
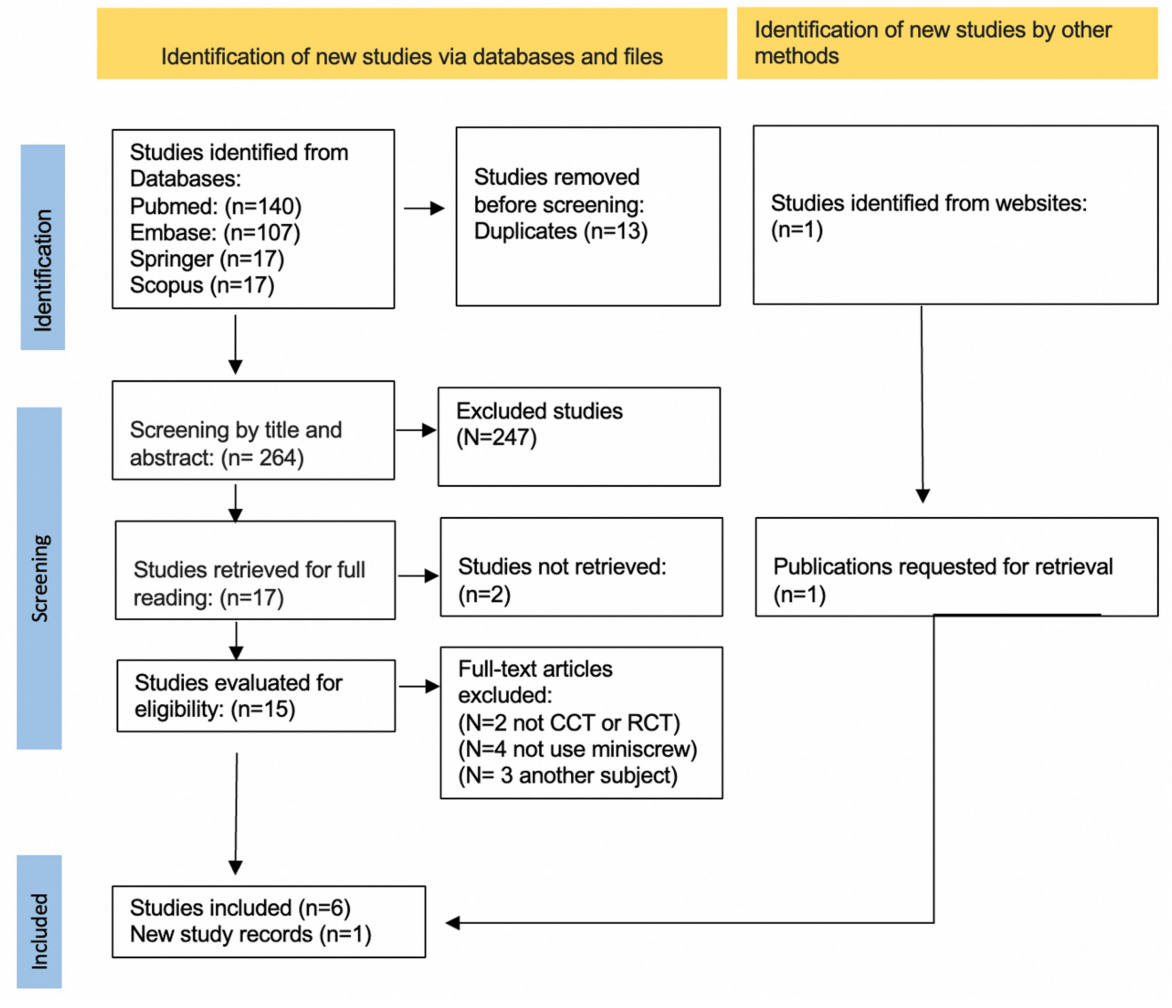
PRISMA flow diagram PRISMA: Preferred Reporting Items for Systematic Reviews and Meta-Analyses

A total of 277 articles were identified in the initial search (Figure 2).

After duplicate removal and abstract screening, 17 potential studies remained for full-text reading; inclusion and exclusion criteria were then applied, and six studies were included for review, with one additional study identified in the updated search. Exclusion criteria in the final phase were: two studies were not RCTs, four did not use miniscrews, and three assessed advantages in cases of respiratory conditions.

Characteristics of the Included Studies

Seven RCTs were included for analysis (Table 1) [11-17].

**Table 1:** Summary of characteristics of included studies and Downs and Black quality assessment scale RCT: Randomized clinical trial; RPE: Rapid palatal expansion; MARPE: Mini-implant-assisted rapid palatal expansion; CH: Conventional Hyrax; RME: Rapid maxillary expansion; B-RME: Band-rapid maxillary expansion; CBCT: Cone beam computerized tomography

Authors, year	Type of study	Objective	Country	Sample size	Assignment	Randomization	Concealment	Blinding	Downs and Black Score	Downs and Black quality
Chun et al. [16], 2022	RCT, Single-blind, parallel, single-center, and two arms	To assess the immediate and short-term skeletal, dentoalveolar, and periodontal effects of RPE and miniscrew-assisted RPE (MARPE) in adolescent and young adult patients Equivalence hypothesis	Korea	40	1:1	Blocks	Sealed opaque envelopes	No	22/28	Good
Garib et al. [12], 2021	RCT, Single-blind, parallel, single-center,	To compare the orthopedic results of CH and hybrid expanders in growing patients. Equivalence hypothesis	Brazil	32	1:1	Simple	Sealed opaque envelopes	Analysis	20/28	Good
Jia et al. [11], 2021	RCT Open-label, parallel, single-center,	To investigate the differences between MARPE and bone expansion to treat transverse maxillary deficiency during the post pubertal growth stage and determine if MARPE is a better alternative Superiority Hypothesis	China	60	1:1	Simple	Sealed opaque envelopes	Not reported	19/28	Good
Lagravere et al. [17], 2020	RCT Single-blind, parallel, single-center,	To compare transverse, vertical, and anteroposterior skeletal/dental changes after treatment in adolescents with skeletal maxillary constriction with the Dresden B-RME appliance with the 4-band RME appliance and an untreated control group. As a secondary purpose, determine if there is a pattern of symmetric or asymmetric expansion in any of these two devices. Equivalence hypothesis	Canada	50	1:1:1	Blocks	Third person indicated the treatment	Analysis	25/28	Excellent
Celenk-Koaa et al. [15], 2018	RCT Single-blind, parallel, single-center, Parallel RCT	To assess and compare dental and skeletal changes with conventional and mini-screw maxillary expansion appliances in adolescents. Non-inferiority hypothesis	Turkey	40	1:1	Blocks,	Sealed opaque envelopes	Analysis	20/28	Good
Gunyuz Toklu et al. [13], 2015	RCT Single-blind, parallel, single-center,	To assess and compare the periodontal, dentoalveolar, and skeletal effects of dental and osseodental expansion devices using cone beam computed tomography. Equivalence hypothesis	Turkey	25	1:1	Simple	Not reported	Analysis	23/28	Good
Lagravere et al. [14], 2010	RCT Open-label, parallel, single-center,	To determine immediate and long-term transverse, vertical, and anteroposterior skeletal and dental changes in adolescents receiving expansion treatment with bone-anchored and tooth-anchored expanders measured on CBCT images.	Canada	62	1:1:1	Simple	Not reported	Not reported	24/28	Excellent

The total sample size across the studies was 309 patients, with ages ranging from 10.8 to 15.1 years. All seven studies reported the success rate of MARPE treatment, which ranged from 68% to 100%. Four studies reported a success rate of 100% [11-13,17]. Random allocation 1:1 to MARPE and Hyrax was carried out in four studies using software-aided simple randomization [11-14] and three used software-aided block randomizations [15-17]. Four of the seven studies reported using opaque, sealed envelopes for concealment [11,12,16], one introduced a third person not involved in the study who indicated treatment at the time of intervention [17], and two studies did not provide this information [13,14]. Blinding of patients and clinicians was not possible due to the nature of the intervention and the need for both to be informed; however, four of the seven studies reported blinding of data analysts [15,13,17], one did not report this blinding [16], and two studies did not clearly specify whether blinding was implemented [11,14]. Quality assessment using the Downs and Black scale was good for six studies [11-13,15-17] and excellent for one [14]. Regarding sample representativeness, there were some weaknesses, as none of the studies reported the proportion of patients who needed the intervention at the institutions where the research was conducted, nor did they report how patients were recruited. Additionally, the quality assessment was affected by the fact that neither the patients nor the clinicians performing the intervention or those in charge of follow-up diagnostic imaging could be blinded.

Risk of Bias in Individual Studies

All studies performed randomization. Regarding allocation concealment, Jia et al. [11], Toklu et al. [13], and Lagravere et al. [14] did not describe a concealment method in sufficient detail, and therefore a definitive assessment of this domain was not possible. Since patients and orthodontists could not be blinded to the intervention, this criterion was evaluated as low risk for all studies, considering that lack of blinding did not generate changes in the outcomes affecting the studies. Chun et al. [16] did not blind outcome assessors, which could introduce some bias in the measurement of outcomes. Jia et al. [11] and Lagravere et al. [14] did not report if assessors were blinded.

**Figure 3:**
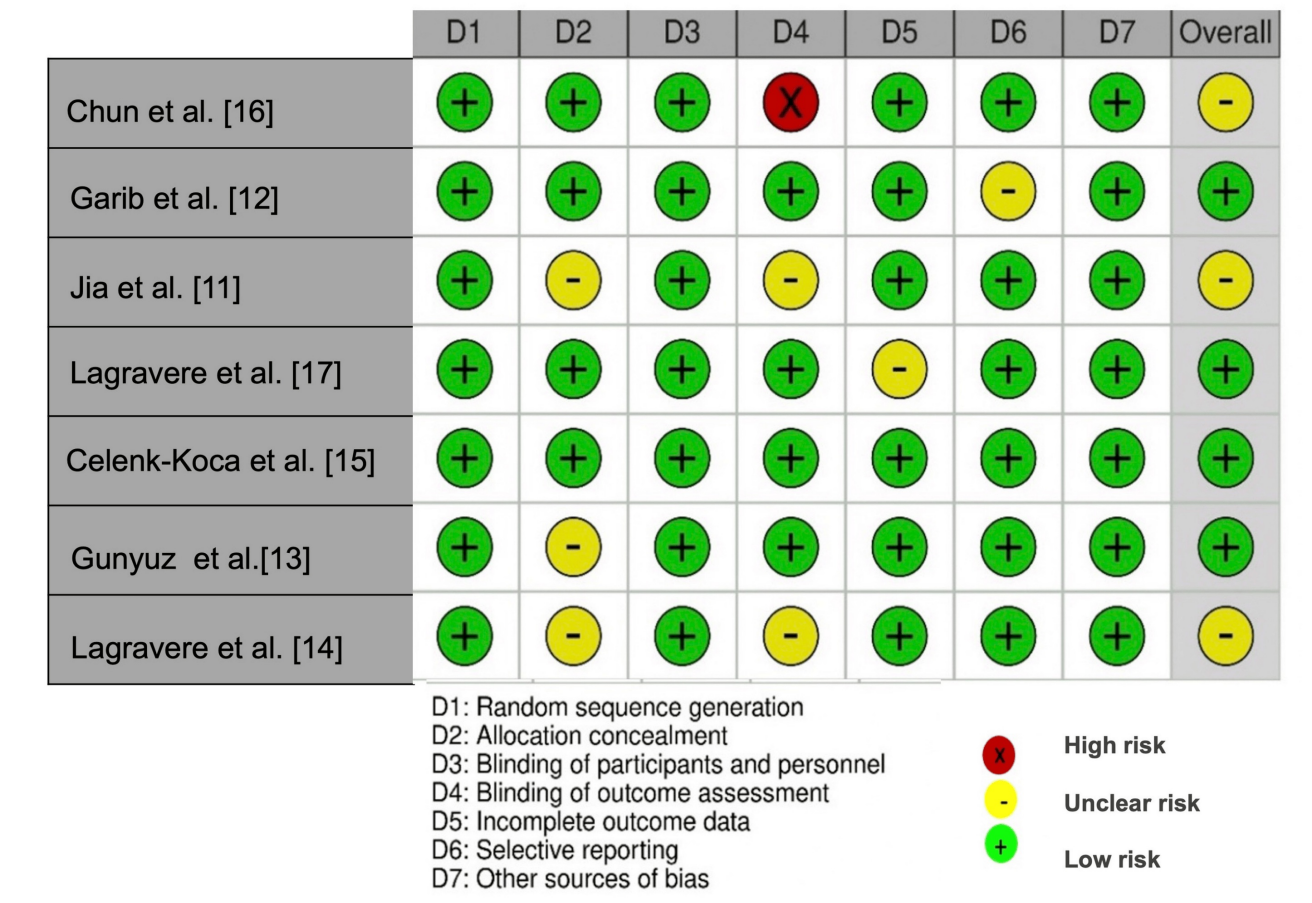
Traffic light graph of bias risk judgments by domains

Regarding bias in the selection of the reported results, the study by Lagravere et al. [14], did not include either baseline reports or a comparison of interventions over time, nor the standard deviation of the mean of skeletal and dental measurements. Therefore, of the included studies, four presented a low risk of bias [12,13,15,17], while three had an unclear risk of bias (Figure 3) [11,14,16].

**Figure 4:**
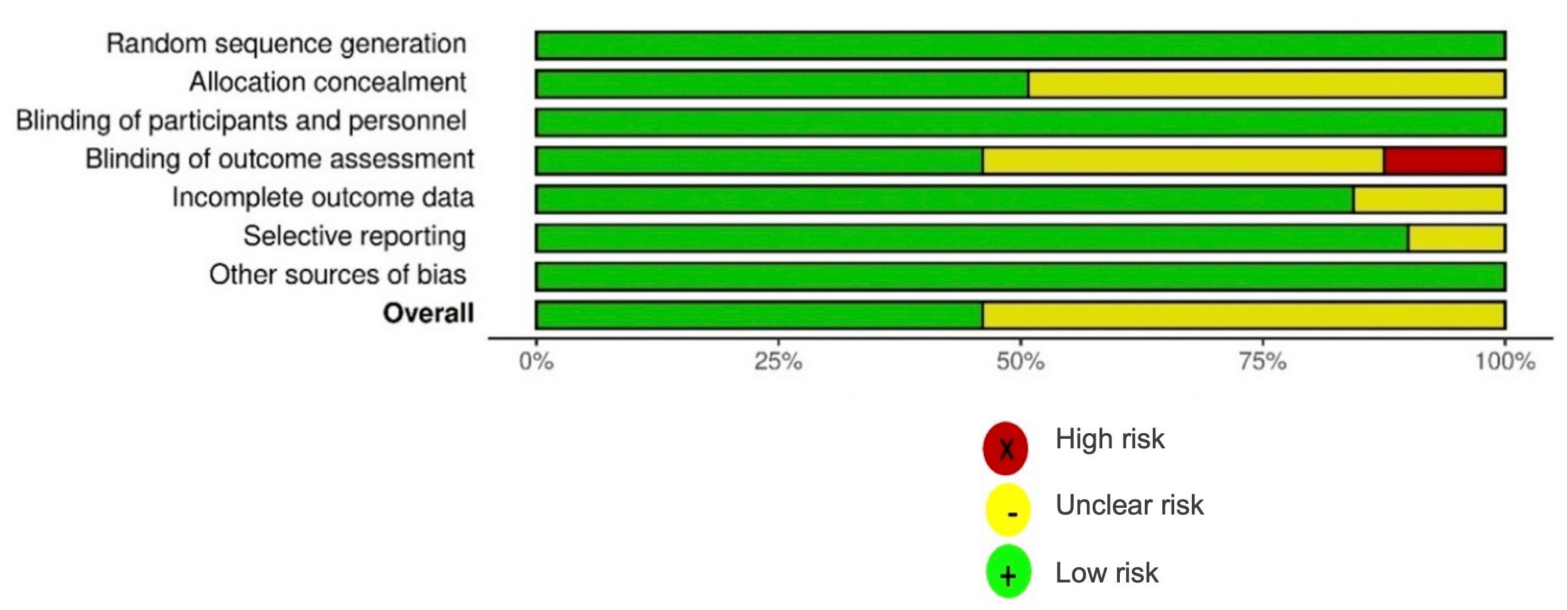
Weighted bar chart of the distribution of bias risk judgments within each domain

Less than 20% of the studies exhibited incomplete outcome data and selective reporting of outcomes, with slightly more than 50% of them having an overall unclear risk of bias (Figure 4).

Results of Individual Studies

**Figure 5:**
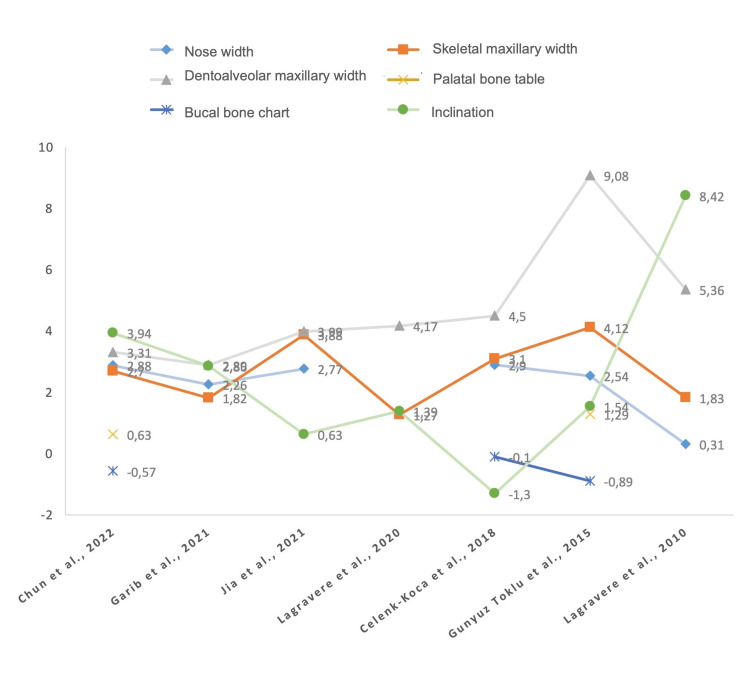
Comparison of averages of transverse skeletal, dentoalveolar, and periodontal changes according to the design of the MARPE presented by each study MARPE: Mini-implant-assisted rapid palatal expansion

Skeletal changes: Skeletal changes were reported in the seven included studies through variations in nasal width and maxillary skeletal width [11-17]. Transverse skeletal expansion assessed through the nasal width was reported in six of seven articles [11-16], with an average increase ranging from 0.31 mm to 2.90 mm for MARPE and from 0.11 mm to 2.46 mm for conventional Hyrax. The difference between the average increase between the two interventions was statistically significant in five studies, which reported greater nasal width increases with MARPE (Figure 5) [11,12,14-16].

The average maxillary skeletal width ranged from 1.27 mm to 4.12 mm for MARPE and from 0.99 mm to 4.59 mm for conventional Hyrax, with the difference between increases being statistically significant in five studies [11,12,15,16,17]. The width achieved with MARPE was greater, with only one study reporting greater maxillary skeletal expansion with conventional Hyrax [13].

Dentoalveolar changes: Measured through dental maxillary width and tooth inclination, dentoalveolar changes were reported in the seven studies, indicating maxillary width increases ranging from 2.89 mm to 9.08 mm with MARPE, and from 2.59 mm to 8.51 mm with conventional Hyrax. The differences were significant in four studies (two favoring MARPE and two favoring conventional Hyrax) [11,14,16,17]. The vestibular inclination of the teeth was measured in the seven studies in different ways, including changes in the longitudinal axis of the tooth with respect to the horizontal plane of the dental arch, change projected to the nasal floor, to the mid-sagittal plane, or in comparison with the longitudinal axis of the contralateral tooth. The inclination of molars was reported in all the studies, while only five of them reported the inclination of premolars. Tooth inclination was statistically significant in four studies [11,13,15,17]. Inclination was between 3 and 9 times greater with conventional Hyrax in three studies, one of which reported significant inclination only for left premolars [13], while another study reported that the inclination achieved with MARPE was negative, i.e., there was a 1.3° palatal inclination instead of buccal inclination [15].

Periodontal changes: Periodontal changes were reported numerically through means and standard deviation only in three studies [13,15,16]. More specifically, these studies reported changes in the vestibular bone plate, but only two of them reported changes in the palatal bone plate. However, six studies mentioned periodontal changes in treated patients [11-16], but they were not clearly reported.

**Figure 6:**
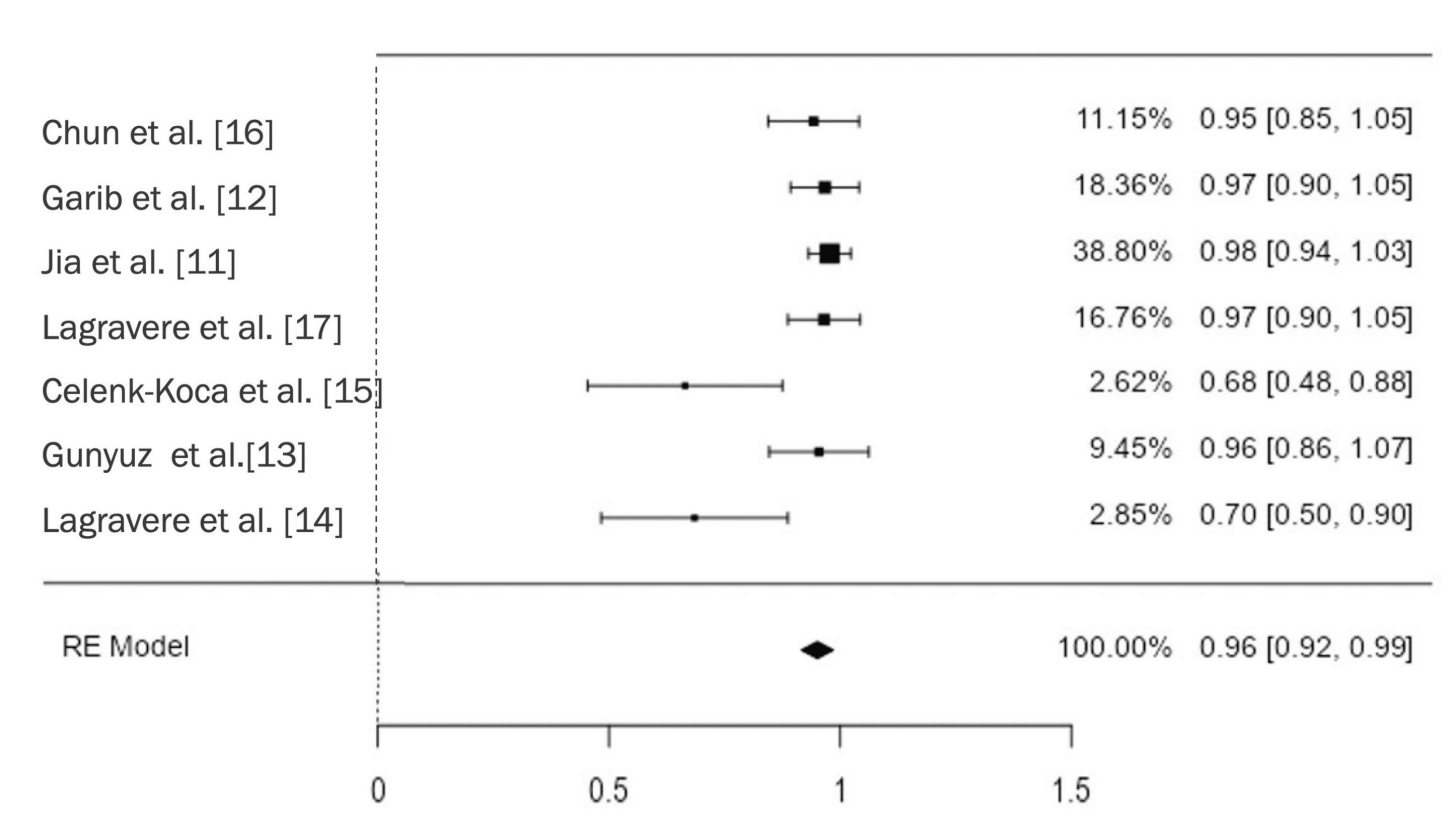
Meta-analysis of proportions for the success rate RE: Random effects

The palatal bone plate increases at the level of the premolars ranged from 0.40 mm to 0.64 mm and at the molars from 0.63 mm to 1.29 mm with MARPE, while it ranged from 1.01 mm to 1.09 mm for premolars and from 0.61 mm to 0.73 mm for molars with conventional Hyrax, but these changes were not statistically significant [13,16]. As regards the buccal cortical bone, reduction at the level of the first premolar ranged from 0.04 mm to 0.45 mm for MARPE and from 0.29 mm to 0.73 mm for conventional Hyrax, with changes being significant in two studies that reported a greater reduction in bone thickness with tooth-borne expanders [15,16]. At the level of the first molars, reductions ranged from 0.10 mm to 0.89 mm in patients treated with MARPE and from 0.24 mm to 0.74 mm with conventional Hyrax. This change was statistically significant in one of the studies, which reported a greater reduction in buccal bone plate thickness with the treatment of the control group, i.e., bone-borne rapid palatal expansion [15]. The overall success rate of the MARPE appliance for rapid palatal disjunction was 96%, with the greatest success rates being reported by Jia et al. [11], Garib et al. [12], and Lagravere et al. [14] (Figure 6).

Design and Anchorage Modifications

When analyzing anchorage and design modifications using meta-analysis and meta-regression, it was found that the greatest skeletal palatal expansion achieved with MARPE was reported by Jia et al. [11], Celenk-Koaa et al. [15], and Gunyuz Toklu et al. [13], with the change being smaller in the second study (Figure 6) [11,13,15]. The greatest skeletal expansions were achieved by Jia et al. [11], and Gunyuz Toklu et al. [13], with overall success rates of 98% and 96%, respectively (Figure 6). These two studies used completely different designs. The first study used an anatomical expander ("s" type) with four tubes and four self-drilling miniscrews with indirect anchorage, achieving an average expansion of 3.88 mm. The second study, on the other hand, used a self-threading hybrid Hyrax appliance with two miniscrews and direct anchorage without dental support, achieving an average expansion of 4.12 mm.

When comparing the designs used in the two studies, it was found that using two miniscrews with direct anchorage [13] resulted in more buccal inclination of the posterior teeth (premolars and molars) with a mean difference of 0.6° and vestibular bone reduction of 1.29 mm, but greater maxillary dentoalveolar width was reported compared to the four-miniscrew design of Jia et al. [11].

The efficacy of the four-miniscrew design with indirect anchorage using bands on the first permanent molars shows a greater success rate and greater maxillary skeletal expansion, greater nasal width, and less dental inclination compared to other designs. However, the authors did not report an assessment of periodontal changes, specifically at the vestibular cortical bone, so this aspect could not be compared in efficacy with the design reported by Gunyuz et al. [13].

Simple MARPE (miniscrews only) with four self-drilling miniscrews results in a greater nasal width, increased maxillary skeletal expansion, and reduced dental inclination. However, the achieved maxillary dentoalveolar expansion is slightly less by a few millimeters on average.

Regarding the miniscrew length, longer miniscrews lead to less nasal width but greater maxillary skeletal expansion, increased maxillary dentoalveolar expansion, and greater dental inclination. However, the difference in average expansion and inclination between short and long miniscrews is not substantial. Therefore, it can be inferred that any length of miniscrew produces similar changes and contributes to the overall success of the treatment. Concerning miniscrew diameter, larger diameters are associated with greater nasal and maxillary skeletal expansion and reduced dental inclination, though maxillary dentoalveolar expansion remains similar across different diameters. Thus, wider miniscrews tend to yield better outcomes.

Performing one activation per day yields improved results in terms of nasal and maxillary skeletal width, as well as reduced dental inclination and maxillary dentoalveolar width. The number of turns, measured in millimeters of daily activation, has minimal effect on the average change in expansion and dental inclination [15-17].

Lastly, total activation, which correlates directly with the number of days of active expansion, shows a directly proportional relationship with average expansion and an inversely proportional relationship with dental inclination. In other words, a greater total activation, and thus a longer duration of active treatment, results in more significant nasal, maxillary skeletal, and maxillary dentoalveolar expansion and less dental inclination.

Discussion

Compared to rapid palatal expansion (RPE) performed with conventional Hyrax appliances, the MARPE technique enables greater nasal width and enhanced maxillary skeletal and dentoalveolar expansion, with less dental inclination at the level of the first premolar. Additionally, MARPE results in a greater increase in the palatal bone plate and less reduction in the vestibular bone plate [11,12,15-17].

To achieve greater skeletal palate expansion, the primary goal of the intervention, while minimizing side effects such as dental inclination, the MARPE appliance typically includes four self-drilling miniscrews of any length but with larger diameters, without the use of additional devices such as mini-implants or onplants. The activation protocol involves activating the appliance once per day, based on the clinician's judgment, since the number of turns was not found to be related to greater or lesser average expansion. The treatment is continued for a longer period until the desired expansion is achieved.

Periodontal changes, which are additional adverse events to dental inclination and pressure on supporting structures, mainly involve excessive loss of the vestibular bone plate. The MARPE technique appears to favor less buccal bone resorption compared to conventional appliances and surgically assisted expansion. According to the results of this study and those of Oliveira et al. [18], Alcin and Malkoç [19], MARPE causes less buccal bone resorption compared to conventional appliances. However, some studies report discrepancies and inaccuracies in evidence. For example, Castrillón-Marín et al. [20] argue that MARPE leads to greater reductions in alveolar bone thickness and bone crest height but note that both periodontal and dentoalveolar complications remain unclear in their study and others. Despite this, Vidalón et al. [21] support MARPE in their review, considering it a superior disjunction system to address these unfavorable clinical situations.

Additionally, the disjunction achieved by the MARPE technique is generally considered stable over time. For instance, Lim et al. [22] reported stable results up to a year after expansion. However, these findings are contradicted by de Marco et al. [23], who reported relapses in the pyriform, integumentary, and bone openings. These conflicting results could not be directly compared to those of this review because not all studies reported follow-up outcomes.

The MARPE appliance design that favors greater disjunction typically involves only miniscrews, without additional devices. However, there is a lack of solid studies to clarify whether a mixed or combined design produces successful outcomes. This study highlights a promising finding, supported by Moon et al. [24], who discuss the lack of consensus regarding the degree of sutural maturation and the relative efficacy of different designs. Due to variations reported in the literature, clinicians often use customized appliances based on their clinical experience and patient needs.

Moon et al. also noted that most authors included in their review found that a four-miniscrew MARPE design provides greater stability and anchorage, leading to successful outcomes in patients with more advanced midpalatal suture maturation [24]. This aligns with the results of the present review and those of MacGinnis et al. [25] who also observed better outcomes with four miniscrews compared to two.

Qualitative results suggest that tooth-borne indirect anchorage offers better control of undesirable posterior tooth inclination. Moon et al. indicate that the use of bands depends on the treatment aim, but that anchorage solely on the first molars can risk affecting the premolars [24]. Therefore, they recommend using anchorage on both molars and premolars if anchorage is to be employed.

Regarding miniscrew length, our meta-analysis revealed no significant difference in the achieved expansion, suggesting that different miniscrew lengths can be used to achieve the desired outcome. Zong et al. explain that miniscrew length should be tailored to the depth of the palatal vault [26]. For patients with constriction and a transverse discrepancy greater than 8 mm, using miniscrews longer than 8 mm may be challenging. Thus, the final decision depends on the professional’s judgment and the patient's clinical situation. Conversely, Wang et al. [27] found that longer miniscrews offer greater stability and achieve better parallelism upon insertion, while Mehta et al. [28] recommend an ideal miniscrew length ranging from 5 to 7 mm of bone insertion at a minimum.

Similarly, Zong et al. [26] and Brunetto et al. [29] suggest that for treating respiratory conditions or problems, it is ideal to use longer miniscrews (greater than 9 mm). Longer miniscrews achieve greater bicortical engagement of the palatal and nasal floors, which reduces stress on the bone and prevents distortion of the MARPE appliance, thereby promoting the expansion of both the midpalatal and nasal sutures.

Regarding miniscrew diameter, larger diameters yield better outcomes, as they result in greater average expansions. This is consistent with the findings of Kapetanović et al. [30], who indicate that larger diameters allow for better adjustment, greater anchorage, and more effective force transmission, particularly in patients with advanced ossification of the midpalatal suture. Additionally, in their in vitro study, Copello et al. [31] found that larger-diameter miniscrews offer greater mechanical stability and retention, reducing the likelihood of miniscrew mobility or pull-out. They also observed increased resistance when combining larger diameters with bicortical anchorage (which involves longer miniscrews) but noted that larger diameters also provide better outcomes with monocortical anchorage.

Regarding activation frequency, performing activation once per day yields better results than twice per day. As explained by Choi et al. [32] and Seong et al. [33], a single daily activation reduces tissue damage and severe pain associated with expansion, as one turn generates significant forces on the circummaxillary sutures. Carvalho Troyano et al. found that this results in maximum stress peaks ranging from 1.5 to 2.0 MPa at the zygomatic process of the maxilla, nasal walls and floor, and vestibular cortical bone when using indirect anchorage [34]. Oliveira et al. report that the MARPE technique generally generates higher tension on the midpalatal suture [35].

This study suggests that performing one activation per day until the required expansion is achieved is the recommended protocol. According to the meta-analyses, a longer treatment duration leads to better outcomes by providing more controlled expansion and reducing the risk of treatment relapse. Excessive tension can cause corresponding excessive compression towards the center of the suture, or lead to excessive expansion, resulting in increased pain and discomfort for patients. Additionally, excessive stress can hinder proper bone and suture remodeling, leading to abnormal osteocyte and fibrocyte activity, connective tissue deposition with hyalinization, or even necrosis of the sutural tissue [36,37].

Limitations and Recommendations

Robust conclusions about the periodontal effects of MARPE are not possible, and these changes cannot be subjected to meta-analysis to determine which design is most favorable for these aspects. More studies incorporating this criterion as an assessment parameter are recommended due to its importance for decision-making.

The heterogeneity of designs, activation protocols, and reference points used to measure skeletal, dentoalveolar, and periodontal changes prevented the identification of calibrated studies on MARPE. This study establishes a baseline for standardizing MARPE design and activation protocols to improve outcomes and allow for more robust statistical meta-analyses. Due to the limited number of high-quality studies with a low risk of bias, meta-analyses had to be conducted separately. Future research should include more randomized clinical trials of high quality to enable quantitative data synthesis incorporating all covariates in a unified analysis.

Protocols should be improved to include specifications not covered in this review, such as miniscrew positioning or the use of alternative techniques like corticopuncture.

## Conclusions

This systematic review and meta-analysis demonstrate that the MARPE technique, especially when using a design featuring four self-drilling miniscrews with daily activation, offers clear advantages over the conventional Hyrax appliance. The findings suggest that MARPE provides superior transverse skeletal expansion, notably in the nasal and maxillary regions, with less undesirable dentoalveolar side effects such as dental inclination and tipping. The use of wider miniscrews further enhances these outcomes, highlighting the importance of anchorage modifications in optimizing treatment success. Clinically, this means that MARPE can be considered a more effective and predictable option for skeletal expansion in non-surgical patients, particularly when maximum skeletal effects and minimal dental movement are desired. This study underscores the critical role of specific appliance designs in achieving optimal skeletal outcomes in rapid palatal expansion. For clinicians, these findings suggest that careful consideration of miniscrew number, width, and activation protocol can significantly improve treatment efficacy, offering a more favorable balance between skeletal and dental effects compared to traditional methods.
